# The Influence of EMG-Triggered Robotic Movement on Walking, Muscle Force and Spasticity after an Ischemic Stroke

**DOI:** 10.3390/medicina57030227

**Published:** 2021-03-02

**Authors:** Patrycja Lewandowska-Sroka, Rafał Stabrawa, Dominika Kozak, Anna Poświata, Barbara Łysoń-Uklańska, Katarzyna Bienias, Anna Roksela, Marcin Kliś, Michał Mikulski

**Affiliations:** 1 Faculty of Rehabilitation, State Higher Vocational School in Nowy Sacz, 33-300 Nowy Sącz, Poland; p.lewandowska-sroka@o2.pl (P.L.-S.); stabrawa@gmail.com (R.S.); 2 Clinical Department, Egzotech sp. z o.o., 44-100 Gliwice, Poland; anna.poswiata@egzotech.com (A.P.); michal.mikulski@egzotech.com (M.M.); 3 Faculty of Rehabilitation, The Jozef Pilsudski University of Physical Education, 00-968 Warsaw, Poland; domuss@op.pl (B.Ł.-U.); katarzyna.bienias@gmail.com (K.B.); 4 Faculty of Automatic Control, Electronics and Computer Science, Silesian University of Technology, 44-100 Gliwice, Poland; Anna.Roksela@polsl.pl (A.R.); m.klis@vp.pl (M.K.)

**Keywords:** stroke, rehabilitation, EMG-triggered, rehabilitation robot, spasticity, mobility, gait

## Abstract

*Background and Objectives*: Application of the EMG-driven robotic training in everyday therapeutic processes is a modern and innovative form of neurorehabilitation among patients after stroke. Active participation of the patient contributes to significantly higher activation of the sensorimotor network during active motor control rather than during passive movement. The study objective was to determine the effect of electromyographic triggering (EMG-triggered) robotic rehabilitation device treatment on walking, muscle force, and spasticity after an ischemic stroke. *Materials and Methods*: A total of 60 participants with impaired motor function and gait after subacute stroke were included in the study. Each patient was randomly assigned to an intervention or control group (IG or CG). All patients, except standard therapy, underwent 1 additional session of therapy per day, 5 days a week for 6 weeks. IG had 30 min of training on the robot, while CG received exercises on the lower limb rotor. The subjects were assessed with Timed Up and Go Test (TUG), Ashworth scale, knee range of motion (ROM), Lovett Scale, and tight circumference at baseline and at weeks 2, 4, and 6. *Results*: For seven parameters, the values credibly increased between consecutive measurements, and for the Ashworth scale, they credibly decreased. The biggest changes were observed for the measurements made with Lovett scale. The average thigh circumference as measured 5 and 15 cm above the knee increased credibly more in the robot condition, as compared to control condition. Additionally, the decrease in Ashworth values over time, although statistically credible in both groups, was credibly higher in the robot condition. *Conclusion*: The inclusion of the EMG-triggered neurorehabilitation robot in the patient’s daily rehabilitation plan has a positive effect on outcomes of the treatment. Both proposed rehabilitation protocols significantly improved patients’ condition regarding all measured outcomes, but the spasticity and thigh circumference improved significantly better in the robotic group in comparison to controls.

## 1. Introduction

Stroke is currently one of the most important health problems in the adult population worldwide, for both medical and social reasons. Almost one third of deaths in the world are caused by strokes [1]. Motor skills are one of the most important areas affected by stroke. Patients may experience a variety of disabilities in different body parts and different hemispheres can be affected. The most common impairments of the early stages of stroke are weakness and paresis, which may lead to a learned nonuse of limbs. Sensory impairments, chronic pain, and immobility of the patient in the early stages after stroke may also contribute to a learned nonuse state [2]. Motor skills impairments are most associated with the reduction in functionality [3]. After stroke, normal muscle activity tone can be also disrupted by neuronal damage. It leads to inappropriate decrease or increase of muscle activity. Spasticity, which is a common issue associated with a stroke, is abnormal muscle tone recognized clinically as resistance to passive muscle stretch, which increases with velocity of stretch. In patients with no functionally useful voluntary limb movement, spasticity can maintain an abnormal resting limb posture leading to contracture formation. In patients with functionally useful voluntary limb movement, inappropriate co-activation of agonist and antagonist muscles can impede normal limb movement [4]. On the other hand, some of the neuronal pathways may remain unaffected after the stroke [5].

Electromyography (EMG)-triggered therapeutic programs create an interaction between the neuromuscular system and robotic device. The electromyographic technique provides more complete information about the patient’s current muscular state (one muscle or the entire muscle group). During rest, bioelectric activity is at the lowest point, and the EMG record shows the degree of muscle stimulation based on the recording of the amplitude and frequency of biopotentials. The main parameters of biopotentials are used as an indicator of relaxation or a state of excitation of specific muscles. Mostly, the devices with at least two channels are used to be able to apply therapy that involves working on antagonist muscles. The patient’s task during the therapeutic session is to control muscle tone for performance with the conditions of the gym, neither using the resistance of the given device, nor in individual work with therapists [6].

Application of the EMG-driven robotic training in everyday therapeutic processes is a modern and innovative form of neurorehabilitation among patients after stroke. Rehabilitation robots with the use of reactive electromyography capture the EMG signal from a patient’s muscles and, on this basis, it assists with a given move. Active participation of the patient contributes to significantly higher activation of the sensorimotor network during active motor control rather than during movement performed passively [7]. Using EMG triggered therapy in rehabilitation of stroke patients provides a possibility to learn how to use these preserved pathways. After a treatment, a patient is able to better control the muscle tone. This accelerates the motor function recovery, which allows for regaining the functional efficiency after a stroke. In addition, the literature proves that the key period in terms of regaining the functional capacity of the body is the first three months after the cerebrovascular incident [7].

Luna EMG provides an electromyographic triggered robot-assisted therapy of upper and lower limbs. In our study, the device was used in patients after stroke to work on the lower limb movement. Luna EMG were detected the intentions of a stroke survivor by means of surface electromyography (EMG) signals located on the hemiplegic side of the lower limb and assisted in the activities of straightening and flexing the knee joint. In addition, while delivering robot-assisted therapy tailored to the individual patient, the system can record signals that may be useful for better understanding stroke recovery [8].

The aim of the study was to determine the effect of electromyographic triggering (EMG-triggered) robotic rehabilitation device treatment on walking, muscle force, and spasticity after an ischemic stroke.

## 2. Materials and Methods

### 2.1. Design Overview

A prospective, randomized controlled two-arm trial design was used: group (1) standard physiotherapy and robotic assisted exercises, and group (2) standard physiotherapy and lower limb rotor exercises. All testing was carried out by the first author who was not blind to participant allocation.

### 2.2. Setting and Participants

Ethical Approval was granted by Bioethics Committee by the District Medical Chamber in Cracow (NR 10/KBL /OIL/2019). All testing took place at “RehStab” Rehabilitation Clinic in Limanowa, Poland. The inclusion criteria were as follows: ischemic stroke, not later than 6 months ago; muscle strength of extensors and knee flexors on the Lovett scale below 3; functional disorders of the lower limb; patient’s condition allows full understanding of commands; and continued/uninterrupted rehabilitation process for 42 days. The exclusion criteria included: cognitive impairment—lack of or poor cooperation between the patient and the therapist; stroke (more than 6 months after the incident); unstable clinical condition; muscle strength of knee extensors and flexors on the Lovett scale greater than or equal to 3; rigid fixed contractures within the lower limb; and significant spasticity (Ashworth scale of 3 and above).

### 2.3. Randomization and Interventions

After written informed consent, demographic information and baseline outcome data had been collected prior to participants getting randomly allocated to a group by a simple randomization. Both intervention groups were then introduced to the allocated program, undertaking each of the program-specific exercises. Both groups participants undertook six weeks of allocated exercise, five times per week, for 90–120 min each session, depending on the patient’s condition. The study participants were divided into two groups: intervention group (A, robot group) and control group (B, control group). In the intervention group (30 patients), rehabilitation was based on individual standard physiotherapy and rehabilitation robot Luna EMG. In the control group (30 patients), rehabilitation was based on individual standard physiotherapy and a use of a lower limb rotor (30 min).

Standard physiotherapy program included:

1. Individual kinesiotherapy (passive and assisted exercises of upper and lower paretic limb, active exercises focused on balance and coordination, breathing exercises, education, or improving gait),
2. Physical therapy (laser therapy, phototherapy, ultrasound, hydrotherapy, pressotherapy), and
3. Classical lower limbs massage.

Luna EMG is a rehabilitation robot specifically designed to aid with the physiotherapy of neurological patients suffering from clinical weakness. It is intended as an all-in-one platform for complex personalized therapy for patients suffering from neurological conditions. It tackles the key problems such as muscle weakness, mobility disorders, gait problems, and range of motion restrictions specifically by automating the process of personalized, motivating physiotherapy based on electromyography, force, and position sensing.

The patient is connected to Luna EMG through extensions—exchangeable mechanical parts that are connected to the patient by straps or by grip. Movement is controlled by a Windows Application from a mobile therapist panel providing a User Interface (UI), patient management, reporting and internet connectivity for the purpose of remote diagnostics and oversight. The device allows us to increase patient muscle force through isokinetic, isotonic, and isometric exercises. The innovative technology “EMG-triggered robotic movement” allows us to work actively with clinically weak patients, even where no movement is visible. Luna EMG detects EMG activity of the muscle and based on that, it provides assistance during the movement. If no movement or activity is present, the device provides passive assistance.

The Luna EMG exercise protocol, which was a combination of CPM (continuous passive movement) with reactive electromyography exercises, consisted of:

- 5 min of CPM of the knee flexion/extension.
- 10 min of EMG-triggered exercise, using the rectus femoris EMG activity to activate the assistance of the device towards extension.
- 10 min of EMG-triggered exercise, using the biceps femoris EMG activity to activate the assistance of the device towards flexion.
- 5 min of CPM of the knee.

Exercises with Luna EMG were conducted in sitting position. Therapeutic procedures were adapted according to the functional state of every person and adjusted individually. All exercises were completed on a one-to-one basis with the first author supervising the sessions.

### 2.4. Outcome Measurements

For the patient’s assessment, several measurement tools were used:

- Timed Up and Go test (TUG): the test is performed from a sitting position. The patient on command gets up from the chair to walk a distance of 3 m, then turns back and sits on the chair at the starting point.
- Ashworth scale (muscle tension examined by the therapist): measurement of resistance during passive knee flexion and extension.
- Tight circumference: 5 cm and 15 cm above the patella.
- Lovett scale assessment for rectus femoris and semimembranosus muscle.
- Range of motion (ROM): knee flexion.

The subjects were assessed with TUG, Ashworth scale, ROM, Lovett Scale, and tight circumference at baseline and at weeks 2, 4, and 6.

## 3. Results

The group covered 45.5% of all 132 patients after stroke who underwent the rehabilitation treatment at the clinic at that time. Sixty-one patients did not meet the inclusion criteria. The total number of patients participating in the study was 71. Eleven patients have been disqualified during the study (3-absenteeism over 10% of training, 5—shorter than 4 weeks, 3—another stroke episode during the research). Sixty participants (27 female) took part in the study. Half of them were assigned to the control group (15 female), and other half to the treatment (robot) group (12 female). Data from the last measurement (sixth week) were not available for 4 participants in the control group and for 8 participants in the treatment group. All 60 patients participated in a 6-week rehabilitation program.

The mean age of patients at the time of research was 66.8 years ± 11.5. The oldest patient was 91 years old, while the youngest was 29. Right-hand side manifestation of the cerebral stroke was observed in 31 (51.7%) cases. Left-hand side paresis occurred in 29 (48.3%) cases. 

**Figure 1:**
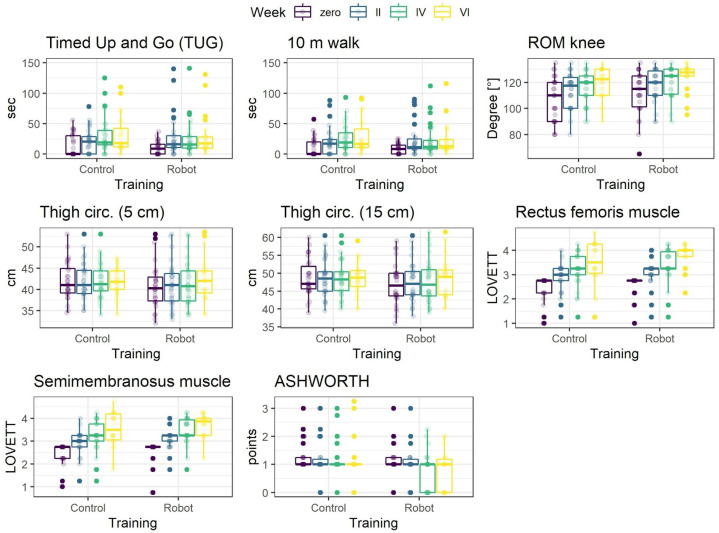
Distributions of parameters separately for training conditions and consecutive measurements (m—meters, ROM—range of motion, circ.—circumference).

Data were analyzed using R 4.0.2 statistical software [9]. Parameters distributions, separately for each experimental condition and measurement time, are presented graphically in Figure 1. Many of the distributions are asymmetrical and numerous deviant observations can be clearly noted in the data. For this reason, to summarize the data medians and median, absolute deviations as measures of central tendency and dispersion are presented in Table 1.

### 3.1. Modeling of the Treatment Effect

To adequately measure the effects of training in both conditions, Bayesian skew-normal multilevel regression with participant specific intercept was used for each parameter. The model allows control for the skewed distributions, unequal numbers of observations per participant without data loss, and for differences in average values of a parameter between participants.

Prior to modeling, the parameters were transformed into Z-scores. Training was coded orthogonally with Robot condition as −1 and Control as 1. The effect of training represents the difference in overall means between conditions. The effect of measurement (week) was treated as an ordered factor and coded using the monotonic effect [10]. This effect assumes that change in a parameter is either monotonically increasing or decreasing (note that the increase/decrease can be nonlinear). Based on the descriptive statistics (Table 1, Figure 1), this is a reasonable assumption for the week effect. Finally, interaction of training and week effects was included. This effect is the most important as it represents the difference in the increase/decrease rate of a parameter between experimental conditions.

In Bayesian statistics, the inference is based on analyzing posterior probability distributions of a model parameters, obtained by integrating likelihood (data) with prior probability distributions. The parameter (e.g., training effect) is said to be statistically credible when 95% credibility intervals (CI) of the posterior distribution exclude zero [11], as a point estimate of the effect means of the posterior distributions are presented.

To approximate posterior distributions of the models, the Markov Chain Monte Carlo (MCMC) sampling procedure was conducted using the brms package [12]. For each reported model, six parallel MCMC chains were used. Each chain consisted of 10,000 samples, with 5000 samples used as a warm up period and every 10th sample recorded, resulting in 3000 recorded samples in total. the sampling procedure was efficient and resulted in well mixed and not autocorrelated chains and unimodal posteriors.

### 3.2. Treatment Effects

**Figure 2:**
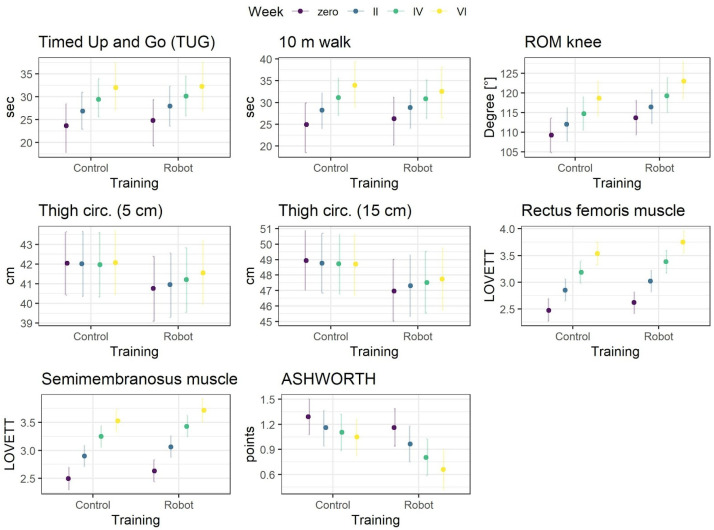
Posterior predictive means (points) with 95% credibility intervals (vertical lines).

Results of the models are presented in Table 2 and posterior predictive means for each treatment condition and measurement are in Figure 2. As a measure of the overall model for Bayesian, the R^2^ is reported [13]. First, the groups did not differ in respect to the overall means of parameters as indicated by absence of credible parameters of treatment effects. Second, for all parameters, the main effect of week was observed, indicating that for seven parameters, the values credibly increased between consecutive measurements, and for the Ashworth, the scale credibly decreased. The biggest changes were observed for the measurements made with the Lovett scale.

Finally, three credible interactions were observed. The average thigh circumference as measured 5 and 15 cm above the knee increased credibly more in the robot condition, as compared to control condition. In the latter, the average circumference was constant over the consecutive measurements. Additionally, the decrease in Ashworth values over time, although statistically credible in both groups, was credibly higher in the robot condition.

In summary, the results indicate that training in both conditions worked, but the training with the robot resulted additionally in a slight but stable increase of thigh circumference and moderately higher decrease in spasticity over time.

## 4. Discussion

The aim of the study was to determine the impact of using advanced technology in combination with conventional therapy on the stroke survivors’ rehabilitation process and its effect on aspects like spasticity, muscle force, or walking. 

EMG-triggered assistive robotic training of neurological patients is an innovative therapeutic approach, which is based on the principles of the usage of robots in rehabilitation. It ensures intensive, repetitive and task-oriented work under the supervision or with the help of a therapist [14]. Research shows that post-stroke motor recovery depends on active rehabilitation by voluntary participation of the patient’s paretic motor system as early as possible in order to promote reorganization of the brain. EMG-based robots have been developed, since voluntary residual motor efforts, to the affected limb, have not been involved enough in most robot-assisted rehabilitation for patients after stroke.

Luna EMG is using the above-mentioned approach and in our study was used to work on the paretic lower limb with patients after a stroke. Currently, there are studies available in the literature using EMG-triggered training in post-stroke patients, but mostly for the upper limb [15]. In addition, there are several types of robotic technology interventions, as multi-joint exoskeletons, to the ones, which are aimed at single joint therapy. Luna EMG robotic device, which was used as an intervention tool for our study, gives a lot of opportunities to train different joints, both in upper and lower limb, but in one axis and has an EMG-triggered assistance, while most of the scientific works are concerned with EMG-triggered resistance training. 

First of all, by looking into the Milot et al. study [16], where they evaluated whether multi-joint functional robotic training would translate into greater gains in arm function than single joint robotic training, we see that no significant difference was noted between multi joint functional and single joint robotic training programs. This challenges the idea that multi joint functional robotic training is superior to single joint robotic training. 

Based on the results of our research, we looked more deeply into the influence of EMG-triggered assistance robotic training technology in combination with conventional therapy on spasticity, muscle force, and walking. 

In our study, both groups have achieved the decrease of the knee spasticity, but the intervention group had significantly higher reduction, compared to the control group. Exercises that promote rapid launch have a positive effect on reducing muscle tone after damage of the central nervous system, while little or no mobilization leads to contractures and an increase in spasticity [17]. The same results were shown in the review of Bertani et al., where 14 randomized controlled clinical trials, two systematic reviews, and one meta-analysis were included to assess effects of robot-assisted upper limb rehabilitation in stroke patients. A modified Ashworth scale was also selected for measuring muscle tone. According to those findings, upper limb rehabilitation using a robot was more effective in improving the restoration of motor functions of the upper limb and was also associated with a decrease in spasticity within the exercised limb in patients with chronic stroke, in relation to conventional therapy. Despite the improvement, no statistical significance was observed, where in our study, this significant difference was noted [18].

Hu et al.’s [19] work is another example of the effective use of robots based on the EMG signal as primary input on spasticity. Researchers evaluated an electromyography (EMG)-driven hand robot developed for post-stroke rehabilitation training. All subjects attended a 20-session training (3–5 times/week), by using the hand robot to practice object grasp/release and arm transportation tasks. Significant reduction in spasticity of the fingers was measured and shown through the Modified Ashworth Scale (*p* < 0.05). In another study of Hu et al. [20], where they investigated the training effects of treatments on the wrist joint of subjects with chronic stroke with an interactive electromyography-driven robot and a robot with continuous passive motion, the interactive treatment reduced spasticity, but also improved muscle coordination. The passive mode training reduced spasticity only in the wrist flexor, but the effect was not long lasting. 

Song et al. [21] were assessing the myoelectrically controlled robotic system with 1 degree-of-freedom, developed to assist elbow training in a horizontal plane with intention involvement for people after stroke. The system could provide continuous assistance in extension torque, which was proportional to the amplitude of the subject’s electromyographic (EMG) signal from the triceps, and could provide resistive torques during movement. After 20 sessions of training, there were also statistically significant improvements in the modified Ashworth scale. The improvement was also noted for the Fugl-Meyer scale for shoulder and elbow, motor status scale, elbow extension range, muscle strength, and root mean square error between actual elbow angle and target angle.

Strength training is commonly considered to be a progressive resistance exercise. However, it should be remembered that any intervention that involves attempted repetitive effortful muscle contractions can result in increased motor unit activity and increase strength [22]. We have analyzed both groups results in that perspective as well. The muscle force of the knee joint was significantly higher in both groups after the rehabilitation process, but the average thigh circumference as measured 5 and 15 cm above the knee increased credibly more after rehabilitation protocol with robotic training included.

Anwer et al. have shown that the combination of isometric exercises with the use of biofeedback increases the isometric strength of the muscle when undertaking training for 5 weeks. Muscle strength in the research group was 23% greater than in the control group at the end of fifth week [23]. The experimental group, which received auditory and visual EMG feedback (which was present also in our training protocol) while exercising, demonstrated significantly greater strength gains than the control group. This shows that it is worth including biofeedback in the patient’s daily exercise, remembering that neurological rehabilitation is a long-term process.

The Son et al. [24] study aims to investigate the idea that an active-resistive training with an EMG-based exoskeleton robot could be beneficial to muscle strength and antagonist muscle co-contraction control after 4-week intensive elbow flexion/extension training. As a result, there was no significant difference in the maximum flexion or extension torque at pre- and post-training. However, the co-contraction ratio of the triceps brachii muscle as the antagonist was significantly decreased after the 4-week training. The active-resistive training with the exoskeleton robot in the older people yielded promising results, showing significant changes in the antagonist muscle co-contraction.

The objective of Song et al.’s [25] study was to evaluate the feasibility of robot-assisted rehabilitation using myoelectric control on upper limb motor recovery. As a result, with the myoelectrically controlled assistive torque, stroke survivors could reach a larger range of motion with a significant decrease in the EMG signal from the agonist muscles. The stroke survivors could be trained in the unreached range with their voluntary residual EMG on the paretic side. After a 20-session rehabilitation training, there was a non-significant increase in the range of motion and a significant decrease in the root mean square error (RMSE) between the actual wrist angle and target angle. Significant improvements also could be found in muscle strength and clinical scales. They concluded that these results indicate that robot-aided therapy with voluntary participation of the patient’s paretic motor system using myoelectric control might have a positive effect on upper limb motor recovery. Basteris et al. [26] also confirmed in their systematic review that stressing active contribution by the patients, e.g., through EMG-modulated forces, may be beneficial to stroke patients. We have measured the ROM of the knee as well, and in our study, both groups have improved significantly in that matter.

Studies carried out by Tsaih et al. [27], in patients with chronic stroke, have confirmed that the use of biofeedback in combination with ankle exercises improved the strength during 6 weeks of muscle exercises. Despite those results, there was no statistically significant improvement in the TUG tests. In our study, where we were focusing on the knee joint in particular, there was improvement of TUG results in both groups, but without any significant difference between them.

A systematic review of robot interventions for gait function improvement in patients with subacute stroke by Ji-Eun Cho et al. showed that some studies indicate a significant difference between the control group and the experimental group that performed general gait training, but other studies did not [28]. Therefore, there is still no clear answer whether the training with the use of rehabilitation robots is more effective for improving gait overall. However, the use of neurorehabilitation robots has its advantages because it reduces the physical load of physiotherapists, allowing them to work with several patients at the same time, which ensures greater throughput and availability of rehabilitation.

EMG-triggered rehabilitation robots can be dedicated to patients who are not able to fully engage in training performed especially at the beginning of the rehabilitation process without the constant help of a physiotherapist. Rehabilitation robots reduce the therapist’s involvement and reduce his physical load, ensuring constant and repetitive training. These benefits have a significant impact on the performance of the physiotherapist’s work in terms of the quality-of-care services [28].

Activation of EMG between antagonist and a pair of muscle agonists around the joint can provide the effect of damping the joint during movement, which in turn can contribute to the accuracy of motion in dynamics movement [29]. Unfortunately, due to a damage caused by stroke, abnormal muscle activation patterns are often observed after it. Movements supported by non-paretic limbs may not be energy efficient, which is directly the case associated with a deterioration in both accuracy and traffic efficiency [30].

## 5. Conclusions

This study indicates that the inclusion of the EMG-triggered neurorehabilitation robot in the patient’s daily rehabilitation plan has a positive effect on outcomes of the treatment. Both proposed rehabilitation protocols significantly improved patients’ condition regarding all measured outcomes, but the spasticity and thigh circumference improved significantly better in the robotic group in comparison to controls. 

Further studies with more entities are needed to create more specific recommendations and protocols in regard to the usage of the EMG-triggered robotic training approach in patients after a stroke. 

## Figures and Tables

**Table 1:** Descriptive statistics.

Parameter	Condition	Week			
		Zero	II	IV	VI
Time up and go	Control	0 (0)	20.5 (20.02)	19 (14.83)	18 (17.79)
	Robot	9 (13.34)	16 (11.86)	15.5 (9.64)	17.5 (12.6)
10 m walk	Control	0 (0)	17 (19.27)	19 (14.08)	18.5 (12.6)
	Robot	8.5 (12.6)	11.5 (9.64)	12 (7.41)	12.5 (8.15)
ROM knee	Control	110 (22.24)	117.5 (11.12)	120 (14.83)	122.5 (11.12)
	Robot	115 (14.83)	120 (14.83)	125 (7.41)	127.5 (3.71)
Thigh circ. (5 cm)	Control	41 (2.97)	41 (3.71)	41.25 (4.08)	41.75 (3.34)
	Robot	40.25 (4.45)	41 (4.45)	40.75 (5.56)	42 (4.08)
Thigh circ. (15 cm)	Control	47 (3.34)	48.5 (5.19)	48.25 (4.45)	48.75 (3.34)
	Robot	46.5 (5.19)	47 (5.19)	46.75 (5.93)	49 (6.67)
Rectus femoris muscle (LOVET)	Control	2.75 (0)	3 (0.37)	3.25 (0.74)	3.5 (0.74)
	Robot	2.75 (0)	3.25 (0.19)	3.25 (0.74)	4 (0.37)
Semimembranosus muscle (LOVET)	Control	2.75 (0)	3 (0.37)	3.25 (0.56)	3.5 (0.74)
	Robot	2.75 (0)	3.25 (0.37)	3.25 (0.74)	3.88 (0.37)
ASHWORTH	Control	1 (0)	1 (0)	1 (0)	1 (0)
	Robot	1 (0)	1 (0)	1 (0.37)	1 (1.48)

**Table 2:** Results of multilevel skew-normal regressions.

Parameter	Population-Level Effects			Ind.-Level Effect	Model		
	Training	Week	Tr.:Week	τ	σ	α	Bayesian *R*^2^
Time up and go	−0.02	**0.09**	0.01	0.06	0.8	11.72	**0.01**
	(−0.12, 0.08)	**(0.03, 0.16)**	(−0.05, 0.06)	(0, 0.14)	(0.73, 0.89)	(7.76, 16.71)	**(0, 0.04)**
10 m walk	−0.02	**0.08**	0.01	0.04	0.75	12	**0.01**
	(0–0.12, 0.08)	**(0.03, 0.14)**	(−0.03, 0.07)	(0, 0.11)	(0.68, 0.82)	(8.07, 16.69)	**(0, 0.03)**
ROM knee	−0.16	**0.22**	0	0.81	0.45	−5.35	**0.09**
	(−0.37, 0.07)	**(0.17, 0.28)**	(−0.05, 0.05)	(0.66, 0.98)	(0.4, 0.51)	(−9.89, −2.52)	**(0.05, 0.17)**
Thigh circ. (5 cm)	0.14	**0.03**	**−0.03**	1.01	0.17	−5.61	**0.03**
	(−0.12, 0.4)	**(0.01, 0.05)**	**(−0.05, −0.01)**	(0.85, 1.22)	(0.15, 0.19)	(−10.97, −0.53)	**(0, 0.11)**
Thigh circ. (15 cm)	0.18	**0.02**	**−0.03**	1.01	0.17	−6.91	**0.03**
	(−0.08, 0.45)	**(0, 0.04)**	**(−0.05, −0.01)**	(0.85, 1.22)	(0.16, 0.2)	(−11.93, −3.4)	**(0, 0.13)**
Rectus femoris muscle (LOVET)	−0.1	**0.51**	−0.02	0.75	0.38	−2.84	**0.32**
	(−0.3, 0.1)	**(0.46, 0.55)**	(−0.06, 0.03)	(0.62, 0.91)	(0.34, 0.42)	(−7.37, 0.57)	**(0.28, 0.37)**
Semimembranosus muscle (LOVET)	−0.1	**0.52**	−0.01	0.73	0.39	−1.26	**0.35**
	(−0.3, 0.11)	**(0.47, 0.57)**	(−0.06, 0.03)	(0.6, 0.89)	(0.35, 0.44)	(−4.54, 1.24)	**(0.3, 0.4)**
ASHWORTH	0.1	**−0.19**	**0.07**	0.86	0.45	−1.97	**0.1**
	(−0.15, 0.34)	**(−0.26, −0.13)**	**(0.01, 0.12)**	(0.72, 1.06)	(0.41, 0.51)	(−8.53, 2.21)	**(0.04, 0.19)**

Credible parameters of the treatment effects are presented in bold.

## Data Availability

Not applicable.
